# Distribution characteristics of potentially toxic metal(loid)s in the soil and in tea plant (*Camellia sinensis*)

**DOI:** 10.1038/s41598-024-65674-1

**Published:** 2024-06-26

**Authors:** Yishu Peng, Haijie Song, Tao Jin, Ruidong Yang, Jing Shi

**Affiliations:** 1 College of Tea Science, Guizhou University, Huaxi District, Guiyang, 550025 Guizhou People’s Republic of China; 2 College of Resources and Environmental Engineering, Guizhou University, Huaxi District, Guiyang, 550025 Guizhou People’s Republic of China; 3 Institute of Mountain Resources of Guizhou Province, Guizhou Academy of Sciences, Guiyang, 550001 People’s Republic of China

**Keywords:** Potentially toxic metal(loid)s, Pollution factor, Enrichment factor, Tea plant, Soil profile, Element cycles, Element cycles

## Abstract

Potentially toxic metal(loid) assessment of tea and tea garden soil is a vital guarantee of tea safety and is very necessary. This study analyzed the distribution of seven potentially toxic metal(loid)s in different organs of the tea plants and soil at various depths in the Yangai tea farm of Guiyang City, Guizhou Province, China. Although soil potentially toxic metal(loid) in the study area is safe, there should be attention to the health risks of Cu, Ni, As, and Pb in the later stages of tea garden management. Soil As and Pb are primarily from anthropogenic sources, soil Zn is mainly affected by natural sources and human activities, and soil with other potentially toxic metal(loid) is predominantly from natural sources. Tea plants might be the enrichment of Zn and the exclusion or tolerance of As, Cu, Ni, and Pb. The tea plant has a strong ability for absorbing Cd and preferentially storing it in its roots, stems, and mature leaves. Although the Cd and other potentially toxic metal(loid)s content of tea in Guizhou Province is generally within the range of edible safety, with the increase of tea planting years, it is essential to take corresponding measures to prevent the potential health risks of Cd and other potentially toxic metal(loid)s in tea.

## Introduction

The presence of potentially toxic metalloids, including arsenic (As), cadmium (Cd), chromium (Cr), copper (Cu), nickel (Ni), lead (Pb), and zinc (Zn), in crops and food has become a hot topic research for their close association with human health. They enter the human body primarily by food and beverage consumption^1^. Excessive amounts of potentially toxic metal(loid) could harm plant, animal, or human health. Some potentially toxic metal(loid)s (e.g., As, Pb, Cd, etc.) are non-essential for metabolism and other biological functions, while others, such as Hg, As, Pb, Cd, and Cr, could disrupt human metabolomics, leading to morbidity and even death^2^. Cd and As are the most toxic elements of the food safety concern due to their relatively easily absorbed into food crops^3^. Cd threatens global food safety and inhibits the growth and development of plants^4^.

Tea is a healthy and delicious beverage that is loved by many. Tea, one of the three major non-alcoholic beverages along with coffee and cocoa in the world, has been enjoyed by most people and become a vital part of the human diet. Tea is a healthy beverage enjoyed worldwide for centuries^5^. Originating in China and spreading to the world in the past 2000 years, tea has become one of the most popular drinks and is in love with more than two-thirds of people globally^6^. Drinking tea could supplement rich mineral elements, amino acids, tea polyphenols, and other beneficial ingredients^7,8^. It could also reduce mental fatigue, lower serum cholesterol, prevent low-density lipoprotein oxidation, and reduce the risk of cardiovascular disease and cancer^9^.

Potentially toxic metal(loid) assessment of tea and tea garden soil is a vital guarantee of tea safety, which is very necessary. Although tea is a widely consumed beverage, it might contain a few potentially toxic metal(loid)s that could harm consumer's health^1^. Researchers have reported that the tea, commercial tea, and tea soup might exit concentrations and possible health risks of potentially toxic metal(loid)s^1,10–14^. The potentially toxic metal(loid) content of tea plants is generally affected by soil conditions. Tea plants, like other plants, absorb the essential and non-essential elements (i.e., potentially toxic metal(loid)s) from the soil during their growth and development^15–17^. Tea plants grow poorly in soil with high potentially toxic metal(loid) concentrations^18,19^. There was a negative correlation between the soil pH and the Cu, Pb, Zn, Mn, Al, and Ni availability of tea plants^20,21^. Understanding the relationship between potentially toxic metal(loid) contents of the tea plant and the soil could effectively reduce the health risks of tea from anthropogenic activities^20^.

Potentially toxic metal(loid)s of tea garden soil mainly come from natural sources and human activities. Compared with other countries, tea gardens or agricultural land in developing countries generally have higher levels of potentially toxic metal(loid)s, with Cd and Hg being the elements with more severe pollution levels, and mainly coming from agricultural activities and industrial activities, respectively^22^. Soil Pb in tea gardens of Anxi County is primarily sourced from the parent material and vehicle exhausts, and soil strontium is affected by anthropogenic sources^23^. Granite/granodiorite and shale were geological sources of heavy metals in soil and tea in this study, and the fertilizer application was a vital anthropogenic source of potentially toxic metal(loid)s^24^. Natural sources accounted for 80.29% and 80.31% of the carcinogenic risk in tea garden soils from south Fujian Province to adults and children, respectively^25^.

So far, studies have been carried out on the relationship between the contents of potentially toxic metal(loid)s (i.e., As and Cd) in different parts of the tea plant^4,26–28^ or in soil at various depths^29^. The Pb content of all parts in the 10-year-old Longjing 43 tea plant was higher in tender branches and roots (absorbing root > secondary lateral root > primary lateral root > main root, bud-producing stems > lateral branch > trunk), while Pb content in new shoots and leaves increased with the increase of maturity (old leaf > mature leaf > one bud five leaves > one bud four leaves > one bud three leaves > one bud two leaves > one bud one leaf)^28^. The order of Cd accumulation in tea seedlings was root > stem > mature leaf > tender leaf^4^. The distribution characteristics of arsenic and cadmium in tea plants showed the law of decreasing from bottom to top: absorbing root > stem or main root > old leaf > young leaf^26,27^. The distribution of As in tea garden profiles (0–10, 10–30, 30–60, and 60–100 cm) in Karbi-Anglong (KA), Cachar (CA), and Karimganj (KG), Assam, and India was investigated^29^. Therefore, it is necessary to systematically and comprehensively study the potentially toxic metal(loid) (i.e., As, Cd, Cr, Cu, Ni, Pb, and Zn) content distribution and relationship of the tea plant different parts and the soil at various depths, to provide suggestions for high quality and safe tea garden management, to effectively reduce the risk caused by too high these elements in tea.

## Materials and methods

### Study area

The Yangai tea farm, built in 1952, is in the southwest suburb of Guiyang City in Guizhou Province, China (26° 23′ 19′′ N and 106° 31′ 27′′ E). This region is situated in the hilly platform of central Guizhou, the gentle slope of the alpine platform, and cloudy and moist air in early spring, which provides good external conditions for the tenderness and excellent quality of tea buds^30^. For example, the altitude and the annual rainfall of this region are respectively within the range of 1200 to 1400 m and 1200 ~ 1300 mm, the yearly average temperature and frost-free period are respective 14.2 ℃ and 247 d, the air is humid, and the ecological environment is excellent. The tea variety grown on the Yangai tea farm is a population variety of early bud and middle leaf type of Shilixiang in northern Yunnan Province of China, which germinates earlier and has denser hairs on the back of leaves than the local varieties^31^.

### Sampling

In mid-August 2018, we collected two representative soil profile samples (YA-1 and YA-2) and different organs of tea plant samples in the Yangai tea farm of Guiyang City, Guizhou Province, China. We collected 55 samples, including 20 soil samples and 35 plant samples^30^. We sampled plant samples from different organs of one tea plant (i.e., trunk xylem, root xylem, trunk phloem, root phloem, flower buds, old leaves, tertiary branches, secondary branches, trunks, and roots at different depths). In the YA-1 sampling site, we took one sample of soil and tea plant root from every 10 cm within the soil depth range of 0 ~ 90 cm and every 20 cm within the soil depth range of 90 ~ 130 cm, respectively. We also collected one soil and tea plant root sample from the hard iron layer of the soil depth 130 ~ 140 cm. In the YA-2 sampling site, we took one sample of soil and tea plant root from every 20 cm soil depth within the range of 0 ~ 120 cm. We also collected a soil sample on the surface of the hard iron layer in the soil depth range of 120 ~ 122 cm and one sample of soil and tea root from the hard iron layer of soil depth 122 ~ 130 cm.

### Complies with international, national and/or institutional guidelines

Experimental research and field studies on plants (either cultivated or wild), comply with relevant institutional, national, and international guidelines and legislation.

### Sample preparation and potentially toxic metal(loid)s determination

After removing the impurities (i.e., large gravel and plant residue), the soil samples were halved by dichotomy and then dried in a thermostatic air-blower-driven drying closet at 30 °C. The plant samples were washed with high-pressure water, rinsed twice with deionized water, and dried in a thermostatic air-blower-driven drying closet at 60 ℃ for 30 min and then at 30 ℃. We refined the dried plant samples with a ceramic knife and ground them with an agate mortar through a 100-mesh nylon sieve. We weighed the dried soil samples and plant samples about 100 g and 0.50 g, respectively. Then we sent them to an accredited laboratory (ALS Minerals-ALS Chemex (Guangzhou) Co. Ltd.) to determine the contents of potentially toxic metal(loid)s (i.e., As, Cd, Cr, Cu, Ni, Pb, and Zn) and Al by inductively coupled plasma atomic emission spectrometry (ICP-AES, Agilent VISTA) and inductively coupled plasma mass spectrometry (ICP-MS, Agilent 7700x).

### Pollution index

Pollution index (PI) is a valuable tool to express the degree of hazard by calculating the ratio of the measured potentially toxic metal(loid) value of the soil to the evaluation standard value of the element^32^. The calculation formula is as follows:

$$ PI = \frac{{C_{i} }}{{C_{0} }} $$

The formula PI, $C_{i}$, and $C_{0}$ represents the pollution index, the potentially toxic metal(loid) content of the soil, and the evaluation standard value of the element, respectively. In this study, the evaluation standard value was from the corresponding risk-based screening values for the soil contamination of agricultural land (pH < 5.5)^33^.

### Enrichment factor

Enrichment factor (EF) is a relative abundance of elements that helps distinguish the source of chemical elements from an anthropogenic source or natural source^32,34–36^. The enrichment factor formula is as follows:

$$ EF = \frac{{\left( {\frac{Me}{{Al}}} \right)_{{{\text{soil}}}} }}{{\left( {\frac{Me}{{Al}}} \right)_{{{\text{crust}}}} }} $$

The neutralization formula $\left( {Me/Al} \right)_{{{\text{soil}}}}$ and $\left( {Me/Al} \right)_{{{\text{crust}}}}$ is the ratio of potentially toxic metal(loid)s to Al in the average content of soil and crust^37^, respectively.

### Bioconcentration factor

Bioconcentration factor (BCF) evaluates the enrichment ability of this element in plants through the ratio of the element content of a plant to its growth medium^34,35,38^. The calculation formula is as follows:

$$ BCF = \frac{{C_{p} }}{{C_{s} }} $$

The formula BCF, $C_{p}$, and $C_{s}$ respectively represents the bioconcentration factor, an element content of the plant, and the element content of the plant growth soil (the average content of this element in the profile soil).

### Translocation factor

Translocation factor (TF) refers to evaluating the ability of the aboveground part of the plant to absorb and accumulate the element through roots by using the ratio of the element content in the aboveground part of the plant to that in the underground part of the plant^39^. Translocation factor assesses the ability to absorb and accumulate potentially toxic metal(loid) through roots^40^. The formula is as follows:

$$ TF = \frac{{C_{Ap} }}{{C_{RP} }} $$

The formula TF, $C_{Ap}$, and $C_{RP}$ represents the translocation factor, the element content of the aboveground part (stem and leaf) in the plant, and the average content of the element in its underground part (root), respectively.

## Results and discussions

### Potentially toxic metal(loid) distribution characteristics of soil profile

There is primarily acidic soil in the research area, and it is suitable for the growth of tea plants. In the soil profiles of the Yangai tea farm, the surface soil is mainly gray-brown or dark brown, while other soil is predominantly light brown, which might be due to the influence of soil humus and soil organic matter. The soil organic matter content and soil pH value of the Yangai tea farm ranges from 0.04 to 8.08 g/kg (the average value being 2.72 g/kg) and 4.61 to 5.81 (the average soil pH value is 5.18), is mainly mineral soil and chiefly acidic soil, respectively. Most soil pH is in the suitable soil pH range for tea plants (4.5 ~ 5.5), and it is aptly for the growth of tea plants^30^.

**Figure 1:**
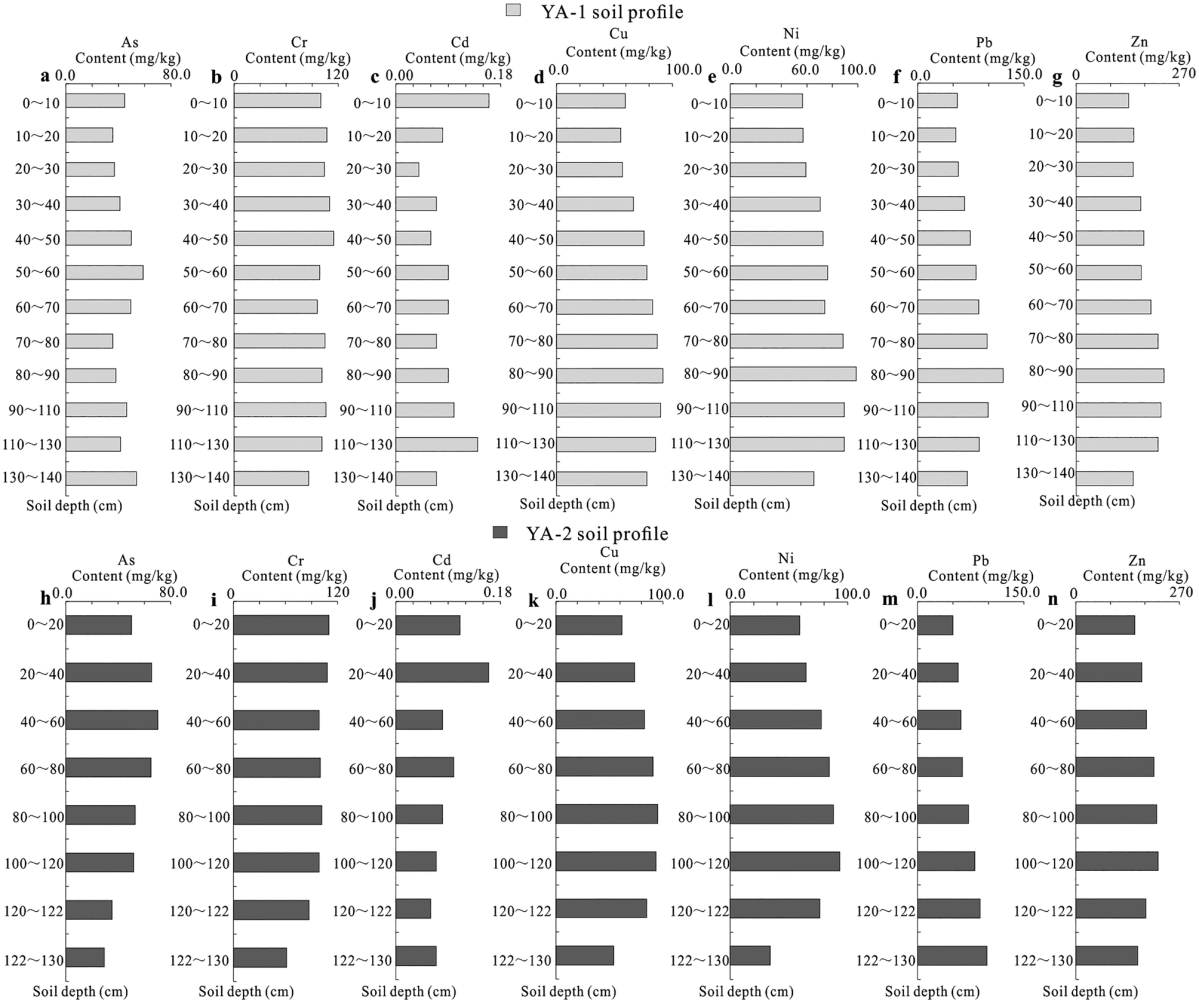
Potentially toxic metal(loid) distribution of soil profile in the Yangai tea farm (**a**, **b**, **c**, **d**, **e**, **f**, and g are respective to the As, Cd, Cr, Cu, Ni, Pb, and Zn content in the YA-1 soil profile, and (**h**, **i**, **j**, **k**, **l**, **m**, and **n**) are respective to the As, Cd, Cr, Cu, Ni, Pb, and Zn content in the YA-2 soil profile).

As shown in Table 1, the average contents of As, Cd, Cr, Cu, Ni, Pb, and Zn in the soil of the Yangai tea farm are 47.58, 0.09, 99.80, 77.13, 73.70, 76.07 and 183.30 mg/kg, respectively, and their content ranges from 29.4 to 70.2, 0.04 to 0.16, 6115, 53.9 to 95.1, 33.9 to 98.8, 50.1 to 120.5, 138 to 230 mg/kg. The average concentrations of potentially toxic metal(loid)s in the soil profile of the study area are in the order of Zn > Cr > Cu > Pb > Ni > As > Cd. As shown in Fig. 1, soil As and Cd contents of the Yangai tea farm were high in the surface and middle layers of the soil profile. Soil Cu, Ni, Pb, and Cr concentrations were high in the middle and lower layers of the soil section. Soil Cr content change was not significant in the whole soil profile.

**Table 1:** Basic statistical parameters of potentially toxic metal(loid)s, pollution index, and enrichment factors in the soil (n = 20).

		As	Cd	Cr	Cu	Ni	Pb	Zn
Contents in soil	Mean (mg/kg)	47.58	0.09	99.80	77.13	73.70	76.07	183.30
	Median (mg/kg)	47.80	0.08	101.00	80.50	75.10	73.50	179.50
	Stdev (mg/kg)	11.29	0.03	11.53	13.66	15.88	18.76	28.18
	CV%	4.21	2.79	8.66	5.65	4.64	4.06	6.51
	Minimum (mg/kg)	29.40	0.04	61	53.9	33.9	50.1	138
	Maximum (mg/kg)	70.20	0.16	115	95.1	98.8	120.5	230
Pollution index	Mean	1.19	0.30	0.67	1.54	1.23	1.09	0.92
	Median	1.21	0.27	0.67	1.61	1.25	1.05	0.90
	Stdev	0.28	0.11	0.08	0.27	0.26	0.27	0.14
	CV%	4.21	2.79	8.66	5.65	4.64	4.06	6.51
	Minimum	0.74	0.13	0.41	1.08	0.57	0.72	0.69
	Maximum	1.76	0.53	0.77	1.90	1.65	1.72	1.15
Enrichment factor	Mean	17.93	0.31	0.68	0.94	0.65	4.19	1.77
	Median	17.56	0.28	0.67	0.95	0.67	3.78	1.71
	Stdev	4.34	0.13	0.09	0.12	0.06	1.55	0.30
	CV%	4.13	2.42	7.79	7.79	10.80	2.71	5.88
	Minimum	11.88	0.15	0.53	0.73	0.55	2.95	1.55
	Maximum	25.68	0.66	0.82	1.25	0.75	10.00	2.96

### Potentially toxic metal(loid) contamination of soil profile

The potentially toxic metal(loid) content of the soil profile in the study area is safe for the growth of tea plants. However, the later tea garden management should pay attention to health risk invigilators and prevention of tea Cu, Ni, As, and Pb. When an element PI of the soil sample is or is not greater than 1, the soil sample is or is not contaminated by the element, respectively^41^. When 1 < PI < 3, 3 < PI < 5, and PI > 5 means that the soil is slightly contaminated, moderately polluted, and seriously tainted by the element, respectively^42^. As described in Table 1, soil As, Cd, Cr, Cu, Ni, Pb, and Zn PIs in the Yangai tea farm range from 0.74 to 1.76 (mean 1.19), 0.13 to 0.53 (0.30), 0.41 to 0.77 (0.67), 1.08 to 1.90 (1.54), 0.57 to 1.65 (1.23), 0.72 to 1.72 (1.09) and 0.69 to 1.15 (0.92), respectively. The average potentially toxic metal(loid) PI order of the soil profile is Cu > Ni > As > Pb > Zn > Cr > Cd. These results indicate that soil Cd, Cr, and Zn in the study area are not polluted, and partly soil samples Cu, Ni, As, and Pb have a slight contamination level. Therefore, the potentially toxic metal(loid) contents of the soil in the research area are safe. However, there should be attention to pollution invigilators and prevention of tea Cu, Ni, As, and Pb in the later tea garden management.

### Potentially toxic metal(loid) sources of soil profile

Soil As, Pb, Zn, and other potentially toxic metal(loid)s of the Yangai tea farm are respectively severe-enrichment, moderate-enrichment, slight-enrichment, and non-enrichment. According to Sutherland^43^ and Chen et al.^44^, EF < 1, 1 ≤ EF < 3, 3 ≤ EF < 5, 5 ≤ EF < 10, 10 ≤ EF < 25, 25 ≤ EF < 50, and 50 ≤ EF correspond to non-enrichment, slight-enrichment, moderate-enrichment, moderate-severe-enrichment, severe-enrichment, relatively severe-enrichment, and extreme-enrichment, respectively. As presented in Table 1, the enrichment factors of soil As, Cd, Cr, Cu, Ni, Pb, and Zn in the Yangai tea farm range from 11.88 to 25.68 (means 17.93), 0.15 to 0.66 (0.31), 0.53 to 0.82 (0.68), 0.73 to 1.25 (0.94), 0.55 to 0.75 (0.65), 2.95 to 10.00 (4.19), and 1.55 to 2.96 (1.77), respectively. Their average enrichment factor order is As > Pb > Zn > Cu > Cr > Ni > Cd. These results show that soil As, Pb, and Zn in the study area are respectively severe enrichment, moderate enrichment, and slight enrichment, and other soil potentially toxic metal(loid) are non-enrichment.

Soil As and Pb have been significantly contaminated by human activities, soil Zn is mainly affected by natural sources and anthropogenic activities, and other potentially toxic metal(loid)s of the soil are chiefly from natural sources. The source of potentially toxic metal(loid) in the topsoil could depend on calculating the EF of soil potentially toxic metal(loid)s^34,45^. When an EF is less than or close to 1 and greater than or close to 3, they indicate that soil potentially toxic metal(loid) is mainly from natural sources and polluted by human activities, respectively^45^. When an EF is between 1 and 3, soil potentially toxic metal(loid) is mainly affected by natural sources and human activities^34^. The average enrichment factors of soil As, Pb, and Zn in the Yangai tea farm are 17.93, 4.19, and 1.77 (Table 1). So, soil As and Pb in the study area has been significantly polluted by anthropogenic activities, soil Zn is mainly affected by natural sources and human activities, and other potentially toxic metal(loid)s of the soil are predominately from natural sources.

### Distribution characteristics of potentially toxic metal(loid)s in various organs of tea plant

As shown in Table 2, the contents of As, Cd, Cr, Cu, Ni, Pb, and Zn in the tea plant of the Yangai tea farm range from 0.018 to 1.420 (average 0.230), 0.036 to 0.457 (0.173), 0.05 to 4.17 (0.65), 1.03 to 154.00 (16.01), 0.57 to 114.00 (12.58), 0.061 to 8.980 (2.041), and 4.6 to 101.0 (21.75) mg/kg, respectively. Their average content order of the tea plant in the study area was the following: Zn > Cu > Ni > Pb > Cr > As > Cd.

**Table 2:** Basic statistical parameters of potentially toxic metal(loid) content, bioconcentration factor, and translocation factor in various organs of the tea plant.

		As	Cd	Cr	Cu	Ni	Pb	Zn
Contents in tea plant (n = 35)	Mean (mg/kg)	0.230	0.173	0.65	16.01	12.58	2.041	21.75
	Median (mg/kg)	0.108	0.135	0.39	4.45	2.59	1.395	13.30
	Stdev (mg/kg)	0.30	0.12	0.80	36.22	29.61	1.88	21.32
	CV%	0.77	1.50	0.81	0.44	0.42	1.09	1.02
	Minium (mg/kg)	0.018	0.036	0.05	1.03	0.57	0.061	4.6
	Maxium (mg/kg)	1.420	0.457	4.17	154.00	114.00	8.980	101.0
Bioconcentration factor (n = 14)	Mean	0.0064	2.1048	0.0090	0.3037	0.2511	0.0356	0.0702
	Median	0.0023	1.5754	0.0046	0.0548	0.0316	0.0255	0.0611
	Stdev	0.01	1.26	0.01	0.59	0.51	0.03	0.06
	CV%	0.84	1.66	0.94	0.51	0.50	1.29	1.26
	Minimum	0.0009	0.8492	0.0016	0.0235	0.0077	0.0097	0.0251
	Maximum	0.0298	5.1061	0.0418	1.9966	1.5468	0.1180	0.2520
Translocation factor (n = 14)	Mean	0.4519	0.8363	0.3460	0.2210	0.2116	0.3869	2.5671
	Median	0.4338	0.6156	0.2761	0.1987	0.1774	0.3203	2.2773
	Stdev	0.41	0.71	0.28	0.13	0.13	0.30	1.71
	CV%	1.11	1.18	1.23	1.67	1.57	1.30	1.50
	Minimum	0.0490	0.2189	0.0681	0.0565	0.0754	0.0226	0.5229
	Maximum	1.6196	2.4692	0.9131	0.4688	0.5189	1.0669	6.2830

**Figure 2:**
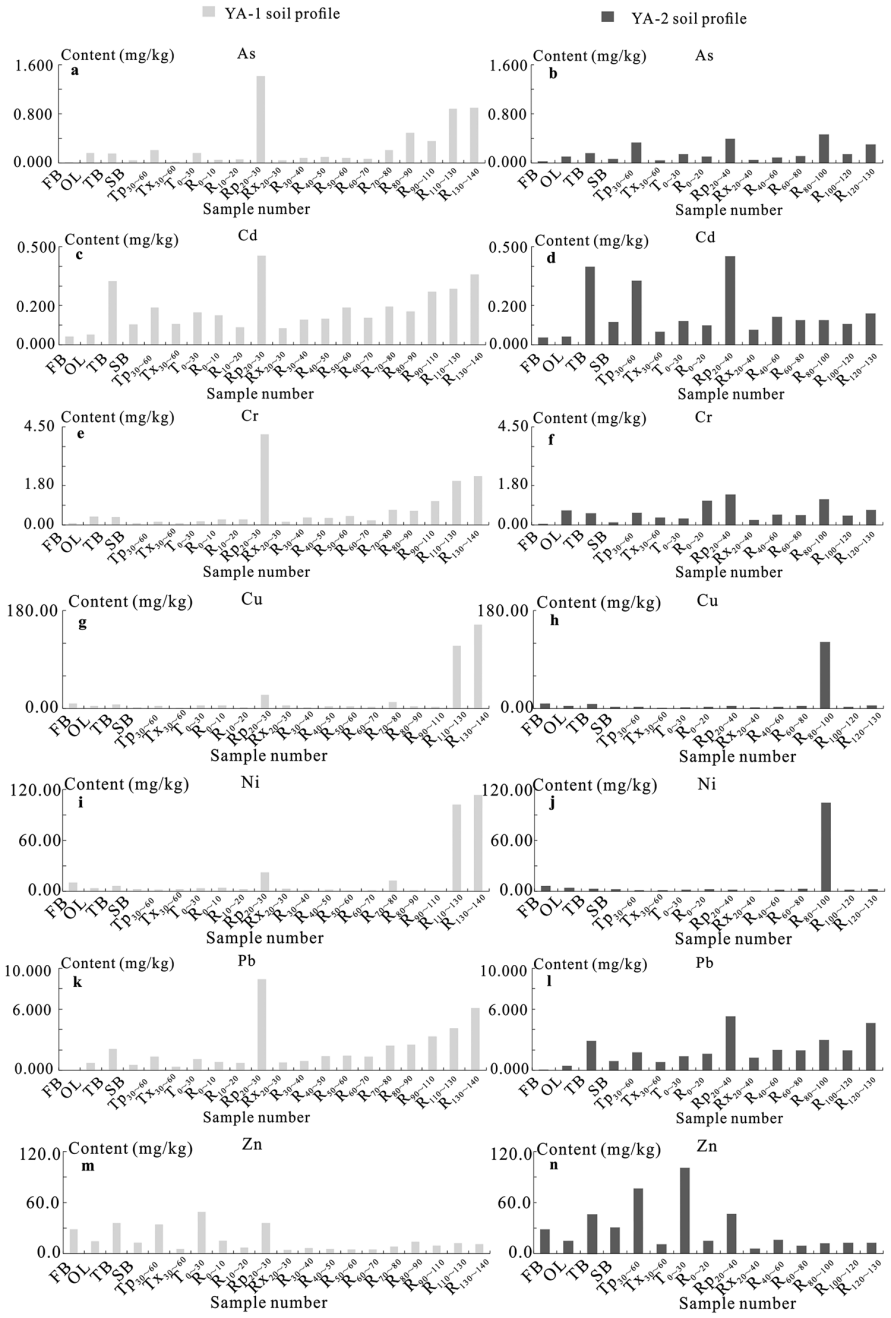
Distribution characteristics of potentially toxic metal(loid)s in various organs of the tea plant in the Yangai tea farm (**a**, **c**, **e**, **g**, **i**, **k**, and **m** are respective to the As, Cd, Cr, Cu, Ni, Pb, and Zn content of various organs in the tea plant of the YA-1 soil profile, and (**b**, **d**, **f**, **h**, **j**, **l**, and **n**) are respective to the As, Cd, Cr, Cu, Ni, Pb, and Zn content of various organs in the tea plant of the YA-2 soil profile).

As shown in Fig. 2, the content distribution of As, Cd, Cr, Cu, Ni, Pb, and Zn in tea stem branches showed third-order branches > secondary branches, phloem > xylem, trunk above ground 30 cm > trunk 30–60 cm above ground (although the content of As, Cd and Pb in trunk phloem 30–60 cm above ground was slightly higher than that in 30 cm above ground, the dry weight of phloem in trunk was much lower than that of xylem. Therefore, its content as a whole shows the trunk from the ground 30 cm > 30–60 cm from the ground. The contents of As, Cd, Cr, and Pb in tea leaves are significantly higher than those in buds, while Cu, Ni, and Zn in tea leaves are higher than those in flower buds.

### Absorption and translocation characteristics of potentially toxic metal(loid)s in tea plant

**Figure 3:**
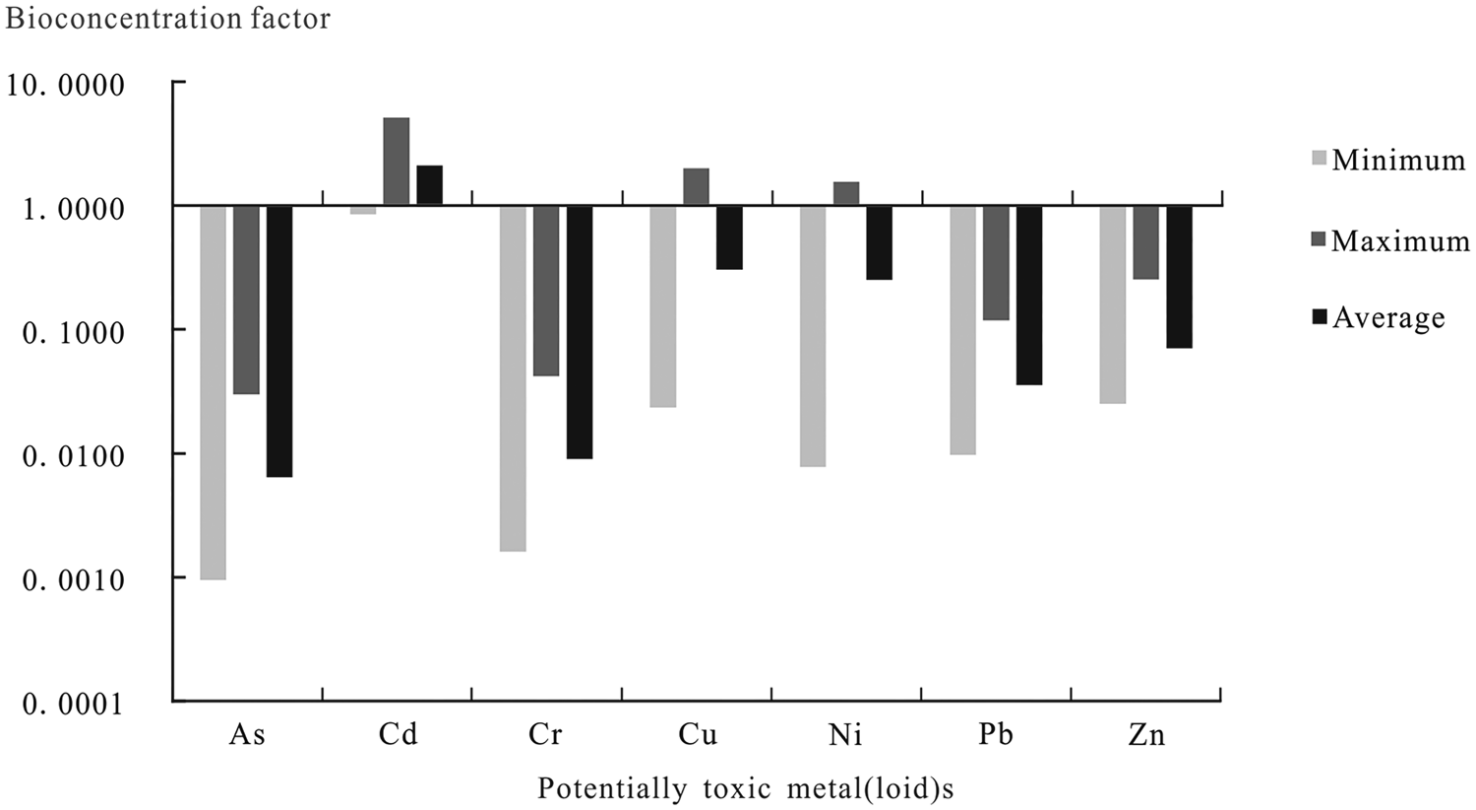
Bioconcentration factor of potentially toxic metal(loid)s in various organs of tea plants in the Yangai tea farm.

The tea plant has a strong capability to absorb Cd and a weak ability to enrich other potentially toxic metal(loid)s. As presented in Table 2, the bioconcentration factors of As, Cd, Cr, Cu, Ni, Pb, and Zn in tea plants in the Yangai tea farm range from 0.0009 to 0.0298 (Means 0.0064), 0.8492 to 5.1061 (2.1048), 0.0016 to 0.0418 (0.0090), 0.0235 to 1.9966 (0.3037), 0.0077 to 1.5468 (0.2511), 0.0097 to 0.1180 (0.0356) and 0.0251 to 0.2510 (0.0702), respectively. The average bioconcentration factor order of potentially toxic metal(loid)s in tea roots in the study area is Cd > Cu > Ni > Zn > Pb > Cr > As. In addition, the average bioconcentration factor of Cd in all organs of tea plants in the Yangai tea farm is more than 1, while other potentially toxic metal(loid) bioconcentration factors are less than 0.5 (Fig. 3). All these show that the tea plant in the study area has a strong ability to absorb Cd and a weak ability to enrich other potentially toxic metal(loid)s.

**Figure 4:**
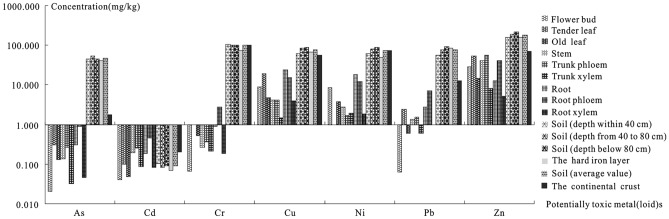
Potentially toxic metal(loid) distribution of the tea plant and soil system in the Yangai tea farm (*note* the data of tender leaves come from the literature^56^ and the continental crust from the literature^37^).

Tea plants may be the enrichment of Zn, the exclusion or tolerance of As, Cu, Ni, and Pb. Potentially toxic metal(loid) accumulators of plants could accumulate in the plant part from low or high background levels^46^. The potentially toxic metal(loid) contents of the soil in the study area are safe on the whole (Table 1), but there should be paid attention to the prevention of As, Cu, Ni, and Pb. As shown in Fig. 4, the soil Cd content of the soil profile in the Yangai tea farm is significantly lower than that in the crust, and the soil As, Cr, Cu, Ni, Pb, and Zn contents are dramatically higher than those in the crust. It shows that the soil in the Yangai tea farm could be rich in potentially toxic metal(loid)s (such as As, Cr, Cu, Ni, Pb, and Zn). At the same time, the As, Cr, and Pb contents of tea plant organs are much lower than those in soil, and the Cd, Cu, Ni, and Zn contents of tea plant organs are similar to those in soil as a whole. It shows that Cd, Cu, Ni, and Zn in various organs of tea plants inherit these element characteristics of the soil, except for As, Cr, and Pb. In addition, growing in a medium with a wide range of potentially toxic metal(loid) content, the potentially toxic metal(loid) exclusion plants could be kept at a constant or low level in their stems(TF < 1), on the contrary, it is potentially toxic metal(loid) enrichment plants (TF > 1)^46^. As described in Table 2, the average translocation factors of As, Cd, Cr, Cu, Ni, Pb, and Zn in the Yangai tea farm range from 0.0490 to 1.6196 (Average 0.4519), 0.2189 to 2.4692 (0.8363), 0.0681 to 0.9131 (0.3460), 0.0565 to 0.4688 (0.2210), 0.0754 to 0.5189 (0.2040), 0.0226 to 1.0669 (0.3869) and 0.5229 to 6.2830 (2.5671), respectively. The average translocation factor order of potentially toxic metal(loid)s in the aboveground parts of tea plants is Zn > Cd > As > Pb > Cr > Cu > Ni. In the same habitat, the translocation factor of Pb, Cd, and Cu in old and young leaves of five tea varieties range from 0.30 to 0.50, 0.06 to 0.37, and 0.20 to 0.41, respectively^47^. Therefore, the tea plant is the enrichment of Zn and the elimination or tolerance of potentially toxic metal(loid)s As, Cu, Ni, and Pb.

### Enrichment and migration mechanism of Cd in tea plants

The tea plant has a unique capability to bioaccumulate Cd and is preferentially stored in roots, stems, and mature leaves. The tea plant suppresses the absorption of Cd when absorbing calcium (Ca) , a sturdier enrichment ability. The bioconcentration coefficient of Cd of tea plants in the Yangai tea farm ranges from 0.8492 to 5.1061, with an average of 2.1048 (Table 2), which is significantly higher than that of other potentially toxic metal(loid)s (Fig. 3). The tea plant has strong bioaccumulation of Ca and sulfur (S)^30^, especially Ca in the flower buds, old leaves, tertiary branches, and trunk stem phloem. In the study area, the Cd content of the third branch, trunk root phloem, and root is significantly higher than that of the bud and old leaves of tea (Fig. 2). The As, Cd, Cr, Cu, Ni, Pb, and Zn contents in the phloem of stems and roots are significantly higher than those in their xylem (Fig. 4), indicating that the tea plants absorb the potentially toxic metal(loid)s from the soil and transport them to tea leaves mainly through their phloem. In addition, the Cd accumulation order of tea plants is root > stem > mature leaf > tender leaf^4^. The distribution of As and Cd in the tea plant shows the law of decreasing from bottom to top: absorbing root > stem or main root > old leaf > young leaf^26,27^. When tea plants are polluted by potentially toxic metal(loid)s, most tea plants accumulate in the roots and migrate to stems and leaves in small amounts^48^. The As, Cd, Cr, and Pb contents of the root and stem in tea plants are relatively high, while other organs are relatively low (Fig. 4). It might be some physiological mechanism that the tea plant inhibits the transfer of these potentially toxic metal(loid)s to its tender leaves and flower buds to avoid the damage of these elements accumulating in these organs. These results indicate that Cd of tea plants is generally preferentially stored in these organs (i.e., roots, stems) rather than tender leaves and flower buds to avoid damage of Cd accumulation. It might explain that tea grown in a high soil Cd content of Guizhou Province has low Cd content. In addition, the buds, old leaves, and phloem of roots and stems of tea plants in the Yangai tea farm have a sturdy capability to absorb and enrich mineral elements such as K, Ca, Mg, S, P, and Mn^30^. The inhibition of Cd^2+^ absorption by CaSO_4_ is mainly due to the competition between Ca^2+^ and Cd^2+^ for ion channels or transporters, inhibits the absorption of Cd by roots and reduces the content of Cd in the cytoplasm, which is beneficial to plant growth and enhances plant tolerance to Cd^49^. These results might be due to the strong bioaccumulation of Ca and S in tea plants. Tea plants not only absorb Ca but also inhibit the absorption of Cd, especially in their tender leaves and flower buds.

In addition, although Cd and other potentially toxic metal(loid) contents of tea from Guizhou Province are generally safe for consumption, prolonged tea cultivation might increase acidity and these elements' availability in the soil to raise these element contents of tea, so it is necessary to take corresponding measures to prevent their potential health risks. Cd is considered one of the most dangerous elements in human health^50^. Exposure to eight potentially toxic metal(loid)s (i.e., Cd, Pb, Tl, Hg, As, Sb, Cr, and Ni) of tea in Guizhou Province does pose no risk to human health through tea intake^11^. Most potentially toxic metal(loid) (such as Cd, Cu, Ni, Pb, and Zn) content distribution of the tea plant is similar to their growth soil (Fig. 4). Mineral element distribution of the tea plant organs mainly originates from the soil and inherits the distribution of these elements in the autochthonous soil^7,30^. As and Cd in various organs of tea plants are chiefly sourced from soil As and Cd^27^. Changes in soil properties and processes can affect food and environmental quality^51^. These show that the tea plant's potentially toxic metal(loid) contents mainly come from the autochthonous soil and inherit their content characteristics. Most amounts of As and Cd are fixed and slowly migrated to aboveground parts of tea plants by absorbing roots and trunk roots when tea plants are contaminated by them^27^. The soil pH value of the tea planting area shows a downward trend with time, which might be that the organic acids secreted by tea plant roots during their growth process increased the acidity of the soil and also increased the potentially toxic metal(loid) bioavailability of the soil^48,52^. Plant roots secrete the organic matter (i.e., organic acid) that could chelate potentially toxic metal(loid) and acidify the rhizosphere to promote their dissolution and root absorption^53^. Therefore, the tea plant cultivated longer may contain higher levels of potentially toxic metal(loid). So, it is necessary to take corresponding measures to prevent the potential health risks of tea, such as plant defense mechanisms and remediation strategies against Cd toxicity (i.e., the use of biochar, mineral nutrition, compost, organic fertilizers, growth regulators, and hormones, as well as the application of phytoremediation, bioremediation and chemical methods^54^, as well as several agricultural practices (i.e., rational tillage, tillage practices, rotation, intercropping and water management)^55^.

## Conclusions

The potentially toxic metal(loid) content of the soil is safe, but tea garden management should pay attention to prevent the health risks of Cu, Ni, As, and Pb in the later stage in the study area. Soil As and Pb contents in the study area are primarily from human activities, soil Zn content is mainly from natural and anthropogenic sources, and other soil potentially toxic metal(loid) contents are chiefly from natural sources.

Tea plants might be the enrichment of Zn, the exclusion or tolerance of As, Cu, Ni, and Pb, and have a sturdy capability to enrich Cd. The tea plant has a strong ability to absorb Cd and preferentially store in its roots and stems. At the same time, the tea plant suppresses the absorption of Cd when it absorbs Ca with a strong enrichment ability. Although tea's potentially toxic metal(loid) contents are generally safe for consumption in Guizhou Province, the increase in tea planting years would lead to rising soil acidity and these elements' availability in soil, which might increase tea's Cd and other potentially toxic metal(loid) contents. Therefore, it is necessary to take corresponding measures to prevent the potential health risks of potentially toxic metal(loid)s in tea.

## Data Availability

The data supporting the findings of this study are available within the article.
